# Tools for Transparency in Central Government Spending

**DOI:** 10.23889/ijpds.v4i1.1092

**Published:** 2019-07-10

**Authors:** C Rahal

**Affiliations:** 1 Department of Sociology and Nuffield College, University of Oxford, Department of Sociology, 42-43 Park End Street, Oxford OX1 1JD

**Keywords:** Civic Technology, Social Data Science, Procurement, Public Administration

## Abstract

Trust in government, policy effectiveness and the governance agenda has rarely been more important than in the opening decades of the twenty first century. For that reason, we herein present centgovspend, an open source software library which provides functionality to automatically scrape and parse central government spending at the micro level. While the design ideals are internationally applicable to any future data origination pipelines, we specifically tailor it to the United Kingdom, a country which is unique not only in terms of its transparency in procurement, but also one which was subject to a parliamentary expenses scandal, years of austerity, and then a volatile political process regarding a referendum to leave the European Union. The library optionally reconciles suppliers and subsequently analyzes payments made to private entities. Our implementation results in scraping over 4.9m payments worth over £3.5tn in value. As a way of showcasing what such a dataset makes possible, we outline three prototype applications in the fields of public administration (procurement across Standard Industry Classifier), sociology (stratification across those who supply government) and network science (board interlock across suppliers) before presenting suggestions for the future direction of public procurement data origination and analysis.

*“I do not think that Ministers understand how little trust there is left.”* Lisa Nandy MP (Wigan) (Lab), House of Commons, 25th March, 2019 [1]

## Introduction

David Cameron’s introduction of new requirements in May 2010 enabled the United Kingdom to lead the world in terms of transparency and ‘Open Data’ [2]: an important ambition to realize given that roughly one in every three pounds spent by the public sector is spent on procurement [3]. The new regime applies first and foremost to central government departments which procure from thousands of suppliers varying in size, ranging from large ‘Strategic Suppliers’ to small businesses. It theoretically allows us to transparently analyze and track longitudinal changes in fiscal policy at a granular level during a period of aggressively targeted deficit reduction.

Procurement data on either individual purchases or payments related to contractual obligations is able to promote government efficiency and effectiveness, as well as empowering citizens with an understanding of the inner workings of the public sector. The provision of such data is mandated across a range of administrative levels (at various financial thresholds), such as central government departments, local authorities [4], smaller local councils [5], and a range of other public institutions such as NHS bodies, emergency services and public transport network providers [6]. At a national level, the publication of information on expenditure over £25,000 is one of the few mandated data-sets that ministerial and non-ministerial departments must provide, with information required on the supplier, the date of transaction, the transaction value, and many other auxiliary fields. The value of providing such ‘Open Data’ is truly enormous, with estimates of the global economic benefit totaling hundreds of billions of US dollars [7]. Applications such as centgovspend which mechanize such data for analysis are essential in realizing the expected progress of ‘Big Data for Policy Making’ [8]. The implementation of these transparency requirements is, however, piecemeal at best.

Various challenges are responsible for a lack of social science literature which utilizes granular public payments despite pioneering efforts by social enterprises, third sector entities (such as the National Council of Voluntary Organisations) and Non-Government Organizations (such as OpenCorporates, Spend Network and the Open Contracting Partnership). Rahal [9] outlines the methodological tools required to map payments from over 300 local authorities to multiple registers, as mandated by the Local Authority Transparency Code, and the Institute for Government [3] uses data from the Spend Network and others to provide a comprehensive description of what is procured, and who from. The most methodologically similar paper to ours [10] develops the Company, Organization Firm name Unifier (CORFU) approach, using it to approximately string match a procurement dataset from Australia from between 2004-2012, with the main difference being that our external reconciliation service normalizes, cleans ‘stopwords’ and expands acronyms on our behalf (steps 1-3 of CORFU), as discussed below. Regarding Public Contracts Ontology, a term frequency-inverse document frequency (TFIDF) based method has been developed to compare the titles of contracts awarded [11]. Hall et al. discuss the use of Semantic Web standards in Open Government Data, with particular regard to data.gov.uk [12].

## Motivation

**Figure 1: Central government debt and Gross Domestic Product in the United Kingdom:**
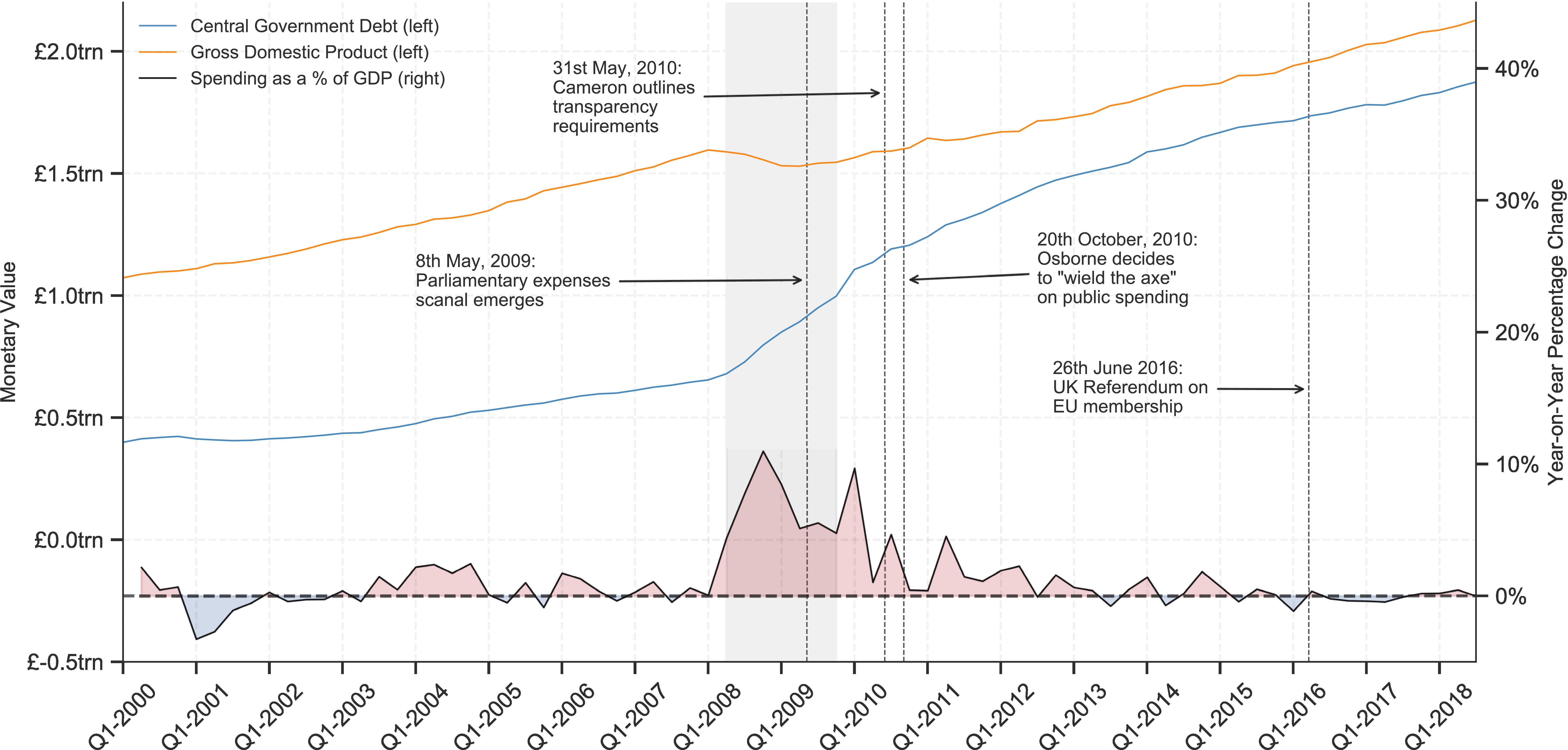
**Figure Notes:** Annotated timeline of UK central government debt, GDP, and a percentage change in debt divided by GDP. All data comes from the OECD. Gray shaded area indicates the recessionary period associated with the ‘Great Recession of 2008-2009‘.

Our motivation can be inferred from two related paradigms: increasingly progressive transparency ideals and trust in governance. The UK leads the world in transparency, frequently topping rankings such as the Open Data Baraometre [13]. This is not just due to the requirements imposed by David Cameron in 2010, but also due to other progressive measures such as the implementation of the Local Authority Transparency Codes [14] and the advocating of the ‘five-star’ approach to Open Data as outlined by Berners-Lee [15]. This increased appetite for transparency is correlated with three key political events which have occured within the same relative period (as annotated in Figure 1). The first is the UK parliamentary expenses scandal; a major political scandal that emerged in 2009, concerning expenses claims made by Members of Parliament over the previous years. The second is the delivery of a highly controversial austerity policy designed to reduce the fiscal deficit, recognized in the academic literature to have negative social and public health consequences [16,17]. The third is the June 2016 referendum to leave the European Union, with the associated parliamentary processes having an eroding effect on trust and belief in democracy.

However, the extremely poor quality of the procurement data at origination creates trouble for aggregation and systematic analysis. It is frequently not provided in the advocated ‘five-star’ format, is subject to severe publication lags1Existing work [3] estimates that just five out of eighteen departments analyzed published more than half of their spending data on time, with a further five failing to publish a single file on time. and is hosted in a fragmented fashion across a variety of different internet sub-domains due to the lack of a centralized Application Program Interface (API). The biggest setback in terms of usability is the lack of unique identifiers (UIDs) which can link and associate individual observations and\or supplying organizations both within and between payments datasets to external sources such as company registers [18]. This lack of inter-operability makes it difficult to build harmonized databases of aggregated payments for systematic analysis: something which centgovspend attempts to facilitate in an accessible and tractable fashion.

## Methods

### Specification: Moving from Motivation to Implementation

The objective of this work is to make better available this unique form of data given the opportunities which it provides for both academic research and civic advocates. Bridging the gap between the raw, noisy, unreconciled and decentalised data through the implementation of our pipeline allows for the building of a conventional ‘flat-file database’ (in a standard delimiter-separated format) familiar to such stakeholders. The simple inputs described below provide details of what (optional) functionality is possible, although the program itself provides no explicit inputs. The software library is a modular set of functions called by a main script (centgovspend.py), all written in Python 3. Modularity in such a commonly utilized language allows extension and customization by other stakeholders. It is operating system independent, contains full logging functionality (through Python’s Standard Library logging module) and accepts a range of command line arguments. The master branch is regularly maintained, and the department specific functions are updated on the first day of each quarter to ensure that content is wrangled from the necessary locations. Content and analysis in this paper is based on the update of the 1st of April, 2019.

### Software Architecture

The first set of functions called by centgovspend.py come from the scrape_and_parse module. It calls a multitude of functions for scraping data from 25 ministerial and 20 non-ministerial departments. The data originates from central government sub-domains split across gov.uk, data.gov.uk and department specific sites.2While the promise of centralization via data.gov.uk is, in theory, encouraging, the lack of implementation leads one prominent analyst to comment of it: ‘We barely use it [data.gov.uk]. I hardly ever use it. I think of the 300 plus scrapers we’ve got set about eight of them scrape to data.gov’ [19] Each department has a dedicated custom function which calls parse_data, iteratively loading the scraped .xls, .xlsx, .ods and .csv files. The procurement data itself is released under an Open Government License (OGL) [20]. We utilize a custom dictionary for harmonizing seven key heterogeneous fields found in each file and a lookup table for dropping all superfluous additional fields. It cleans rows and columns which contain above a threshold percent of non-null values and converts data to the appropriate type (i.e. ‘amount’ to float, ‘supplier’ to string, and so forth) and drops payments below £25k (to harmonize across departments). Functions from the evaluation.py module then evaluate the data acquisition stage while dropping redacted suppliers and anomalous entries (such as where the supplier’s identity equals ‘various’ or similar). At the time of writing, the cleaned version of the data-set contains information from 2,499 files across 854k rows of data worth over £1.318tn.

**Figure 2: Process Mapping of centgovspend:**
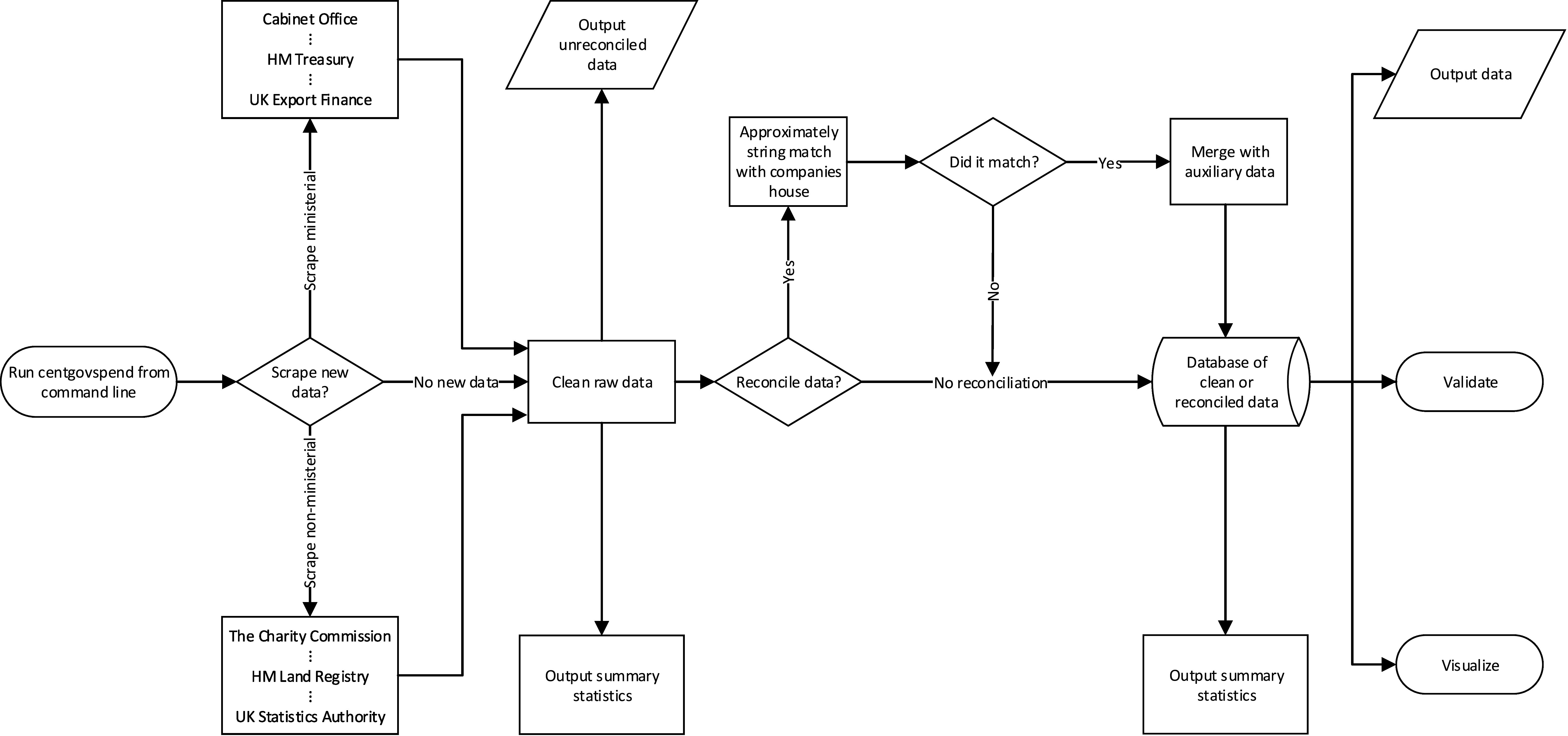
**Figure Notes:** Rhombus relate to decision nodes. Rectangles are processes. Ellipses indicate Start/End. Parallelograms indicate data out. The horizontal three-dimensional cylinder indicates a database.

The program then optionally takes the cleaned, merged tab-delimited output from the scrape_and_parse function calls and attempts to reconcile the unique supplier names with Companies House identifiers via the Elasticsearch based OpenCorporates Reconciliation API. We provide functionality to call this simple REST API outside of the more commonly utilized OpenRefine, waiting three seconds per request. We provide a function (clean_matches) which allows two types of matches to be implemented from the API returns. The first represents an extremely conservative automated matching algorithm (which we term ‘automated_safematch’), which automatically accepts all returns where the first match score returned from the API is greater than 70 and the second is at least ten points lower. This prevents uncertain matches in absolute and prevents potentially ambiguous matches where multiple (alpha-numerically similar) alternatives exist. The second method (termed ‘manual_verification’) allows users to manually verify each match with a score above 0 through direct user input with scores above 70 and 10 points greater than the alternative being automatically accepted. Finally, the company numbers corresponding to the reconciled company names (which are UIDs) are then used to build three auxiliary data-sets for analysis by calling various methods of the Companies House API. We build data-sets related to basic data, company officers and Persons of Significant Control (PSC), adhering to the API ratelimits (600 requests per five minutes) with the ratelimit function decorator. While the library contains code to build in locally ran Elasticsearch based reconciliation functionality with normalized inputs (should the OpenCorporates API cease to be freely available), the code defaults to the OpenCorporates API which has been developed for this specific purpose. This is visualized as a Process Map in Figure 2.

### Evaluation, validation and diagnosis

Figure 3 is designed to evaluate, validate and diagnose the output of the library. Figure 3a presents an overview across time, utilizing the time-stamp field within each parsed file. It shows a fairly consistent degree of coverage, although it highlights a variance in the magnitude of payments recorded consistently over time. Figure 3b compares payments within the financial year of 2017-2018 with the departmental budgets observed in official government documentation (the Public Expenditure Statistical Analyses 2018 – PESA [21]). It highlights that while the ratio of payments aggregated by centgovspend to PESA is close to one, some departments have a ratio slightly above (Department for Education: ratio of 1.05) and somewhat below (Department for Work and Pensions: 0.13). While the un-observable property of this data originating process means we can never be sure of the reasons for this variance, we hypothesize that it is likely due to redactions (for ratios below one) and repayments across financial years (for ratios above one). As with related work, our figures do not correspond perfectly, demonstrating the difficulties involved in generating value from the patchy data available [3]. Figures 3d-3e outline the distribution of results from the reconciliation process.

Figure 3: Evaluation, validation and diagnosis of centgovspend outputs**Figure Notes:** Figure 3a details the coverage of centgovspend over time, with the gray shaded area indicating a time before the implementation of transparency requirements. Figure 3b visualizes government allocations from PESA (financial year 2017-2018), matched with data collected by centgovspend. Figure 3c is a simple count of files parsed by centgovspend. Figure 3d shows the distribution of the highest scores returned from the OpenCorporates reconciliation API. Figure 3e shows a scatter of the first and second highest scores for each unique supplier.

**(a) Longitudinal coverage of centgovspend:**
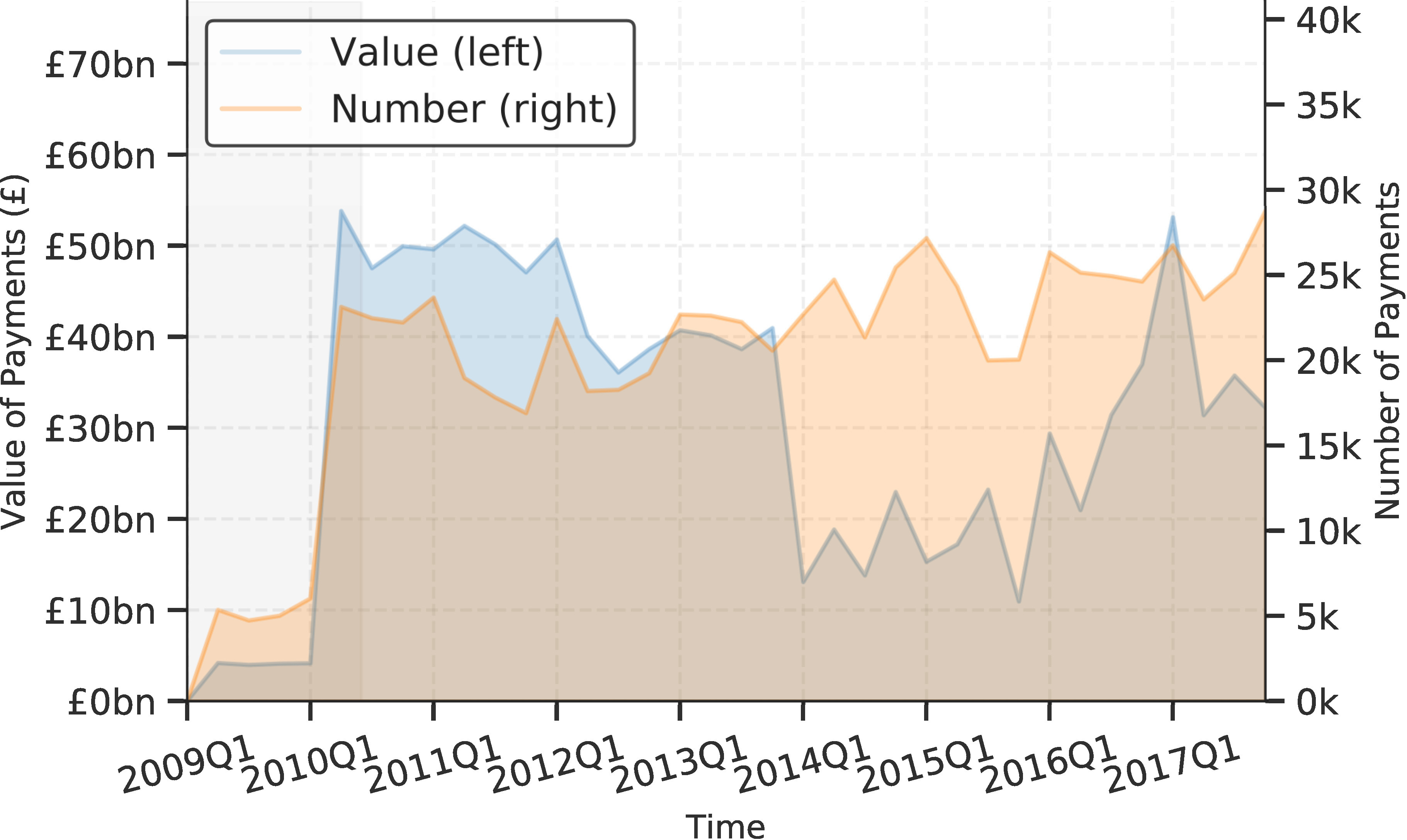

**(b) Comparison with allocations in PESA:**
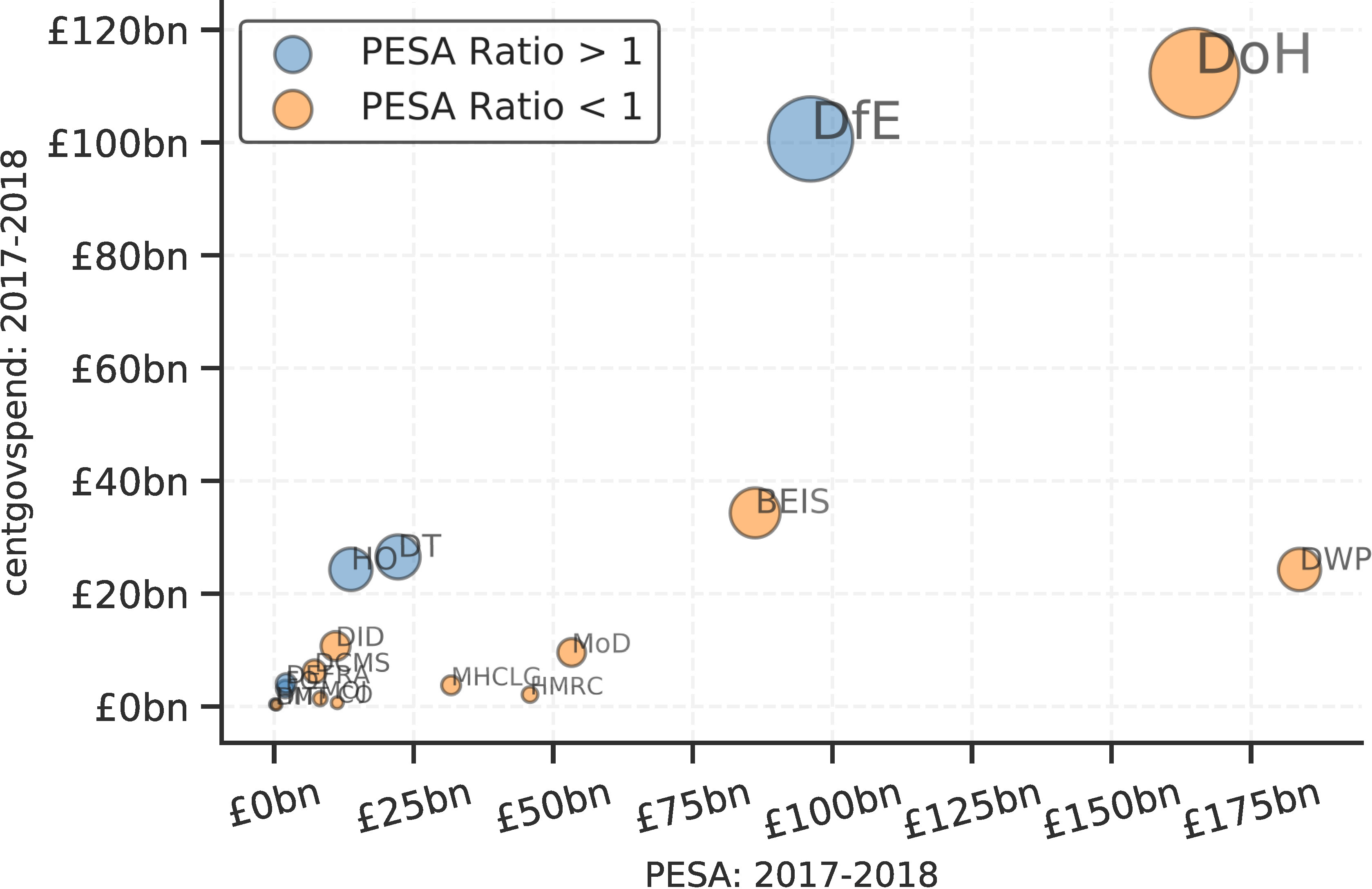

**(c) File counts across departments:**
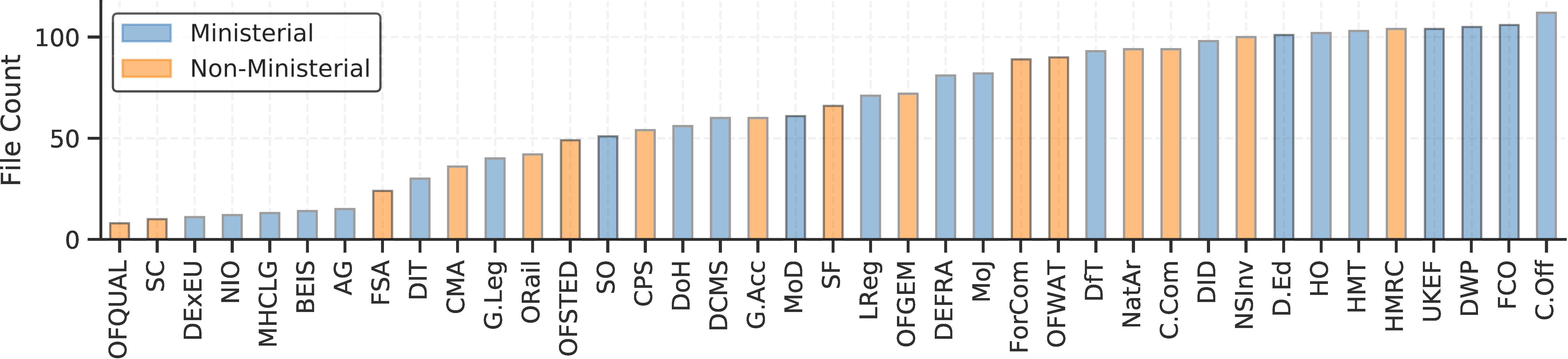

**(d) First scores and cutoffs:**
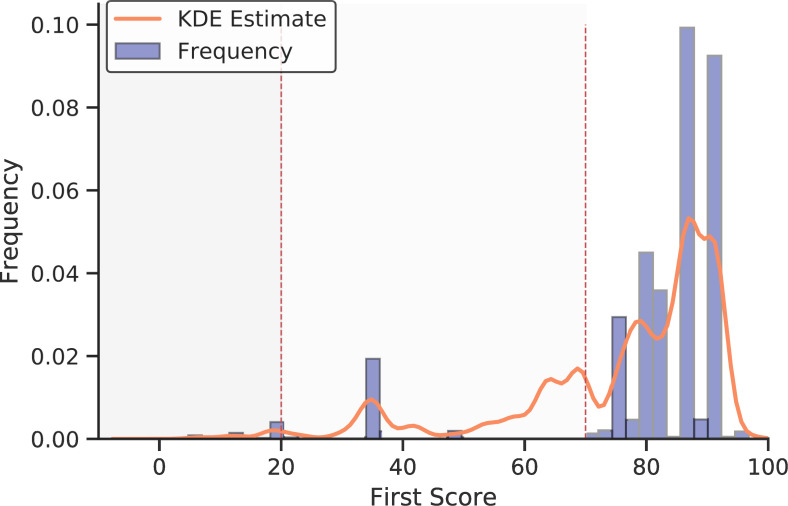

**(e) First and second scores and cuttoffs:**
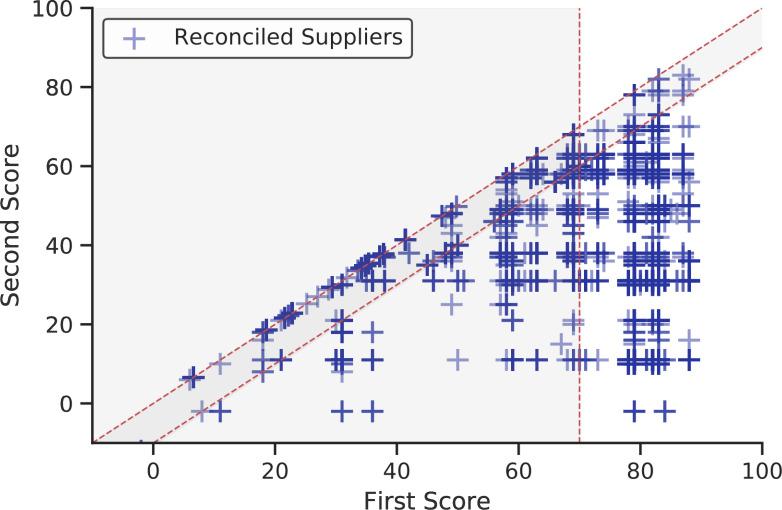

### Software functionalities

The main script (centgovspend.py) accepts multiple inputs, outlined below. We provide an example execution:

python centgovspend ministerial cleanrun noreconcile

assimilates a dataset of spending by ministerial departments (having erased previously assimilated data) which is not reconciled. Optional command line arguments for centgovspend:

- ministerial: only scrape and parse ministerial depts (default = on).
- nonministerial: only scrape and parse non-ministerial depts (default = on).
- cleanrun: delete all previously scraped data (default = off).
- noscrape: dont scrape any new data (incompatible with cleanrun, default = off).
- noreconcile: no reconciliation with OC or CH (default = off).

## Illustrative Results

**Figure 4: A decomposition across departments and aggregated SIC codes:**
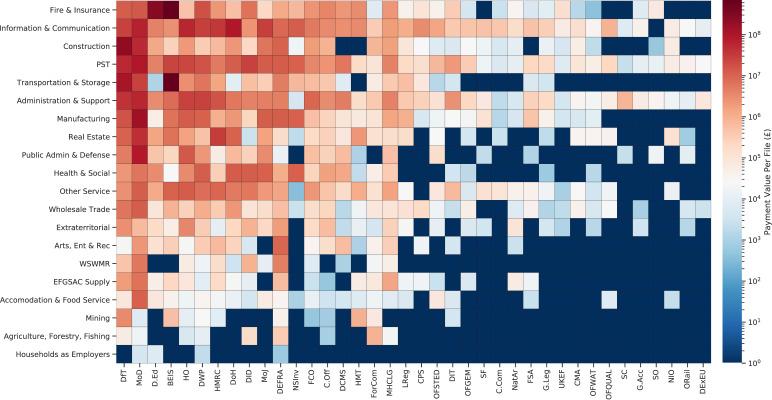
**Figure Notes:** Standard Industry Classifiers (y-axis) are grouped into higher order aggregate categories, ordered by magnitude of summed total spend. Department acronyoms (x-axis) also ordered by magnitude of summed total spend.

### Analysis across Standard Industry Classifiers

The first illustrative example which we provide is a decomposition between departments and across Standard Industry Classifier (SIC) codes, as seen in Figure 4. Such an analysis (and further expansions are provided in an accompanying Jupyter Notebook) allows both departments and potential contractors to better understand the dynamics of the distribution of spending across industries with huge potential for efficiency savings to be generated. A simple tabulation of suppliers, conditional on the thresholds used in the reconciliation approach, would allow a simple analysis of which departments procure how much from the private sector.

### Social stratification across officers and control

The second illustrative example pertains to a sociological application in the form of analyzing the social stratification of specific facets of companies and company operation and ownership. Examples presented here include age distributions in Officer and Persons of Significant Control composition (Figures 5a-5b) and their nationalities and countries of residences (Figure 5c). An accompanying module of centgovspend provides functionality to analyze the subset of (de-identified) reconciled government suppliers with the entire population of the Companies House registry. While a full analysis is beyond the scope of this paper (and additional analysis is undertaken in the supplementary material), important conclusions to be drawn are that the subset of Officers and PSC suppliers which are supplying central government are older and less internationally diverse than the population of Officers and PSC within Companies House.

Figure 5: Officers and PSC in suppliers and the entire population of Companies House**Figure Notes:** Figures 5a-5b show histograms with overlaid kernel density estimates of the age distribution in the entirety of Companies House and reconciled government suppliers for Officers and PSC respectively. Figure 5c compares the number of UK and non-UK nationals and UK and non-UK countries of residence across both Officers and PSC, comparing the entirety of Companies House and reconciled government suppliers.

**(a) Age distribution of company Officers:**
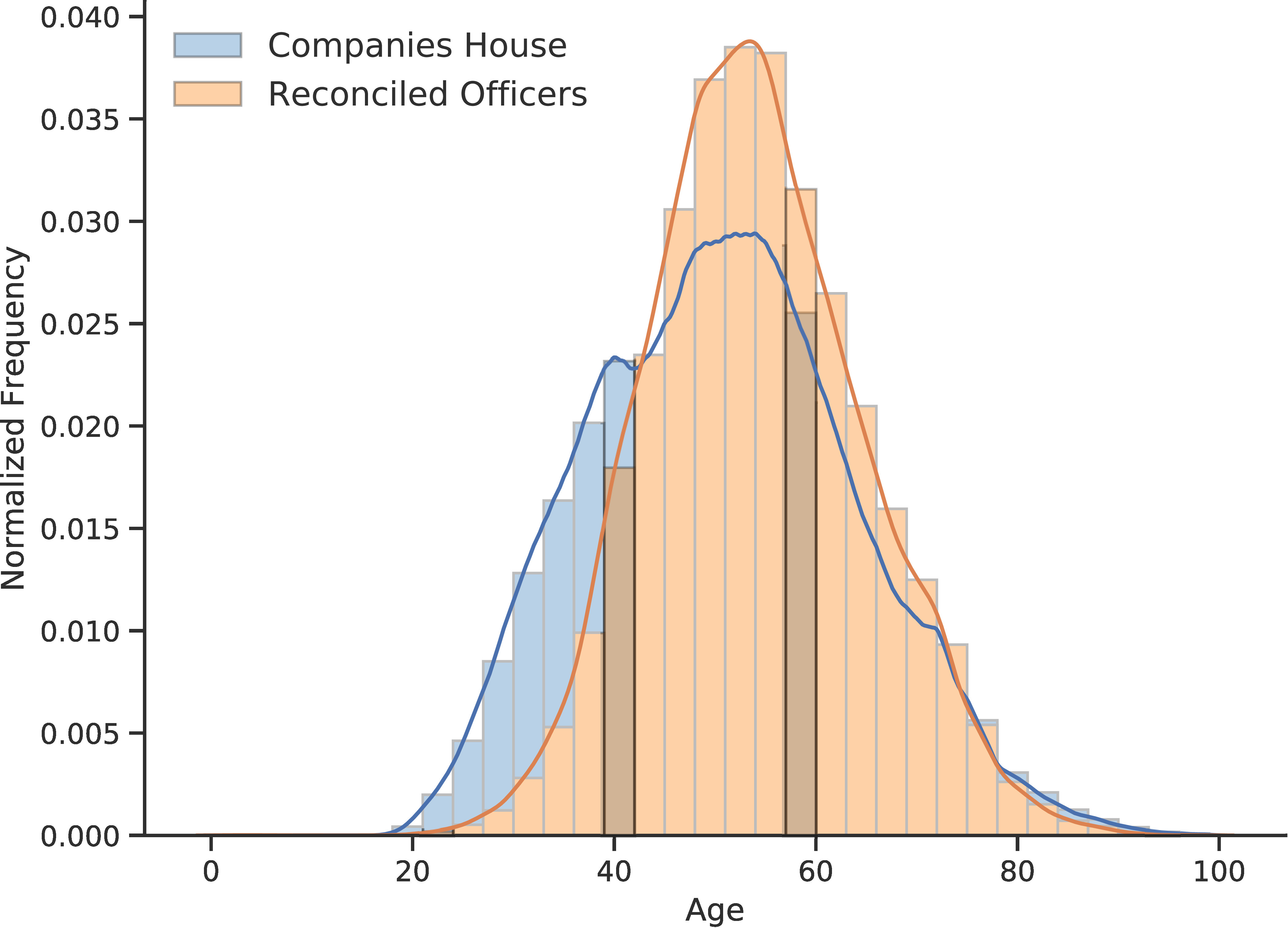

**(b) Age distribution of PSC:**
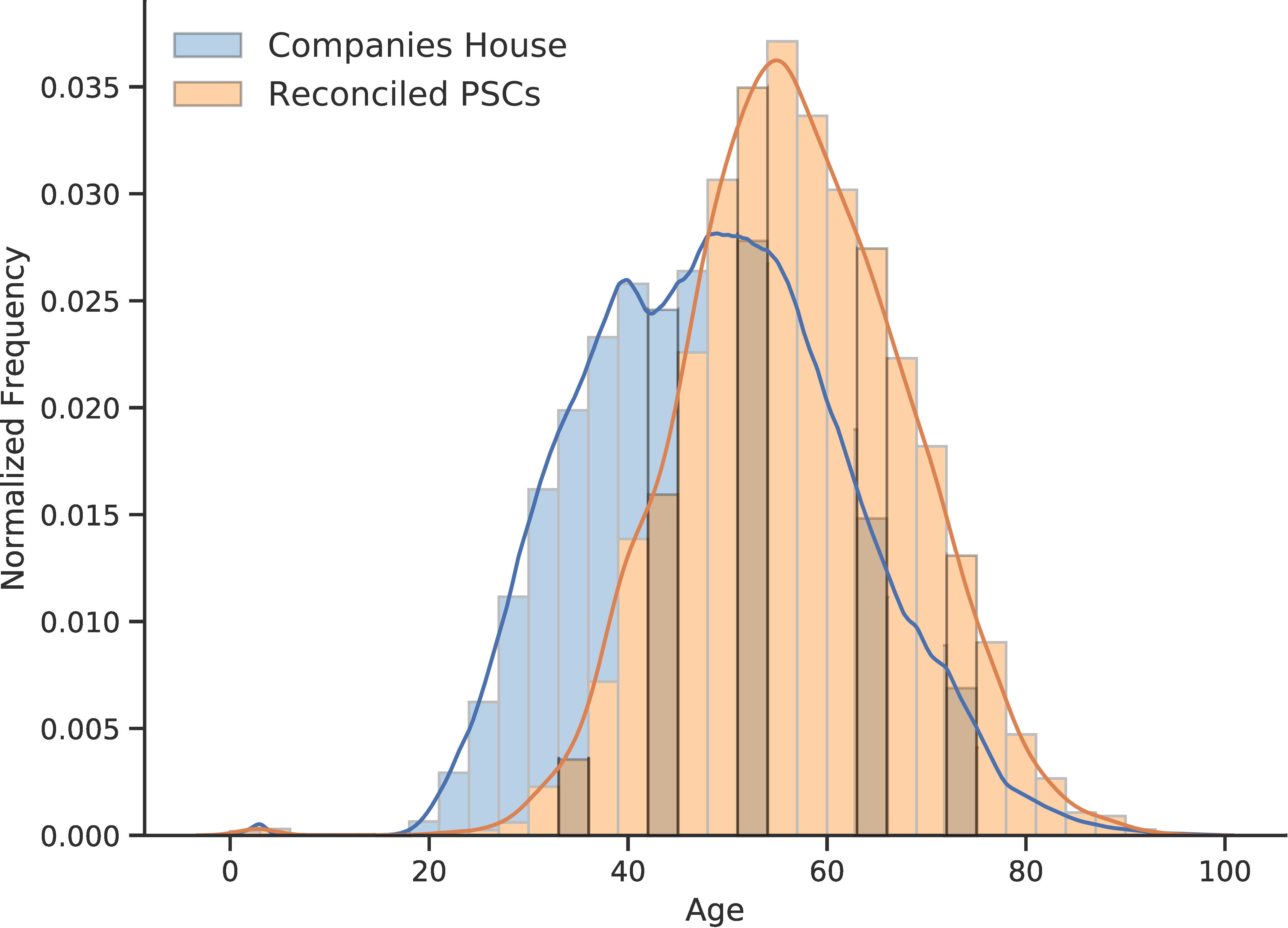

**(c) Nationality and country of residence of Officers and Persons of Significant Control:**
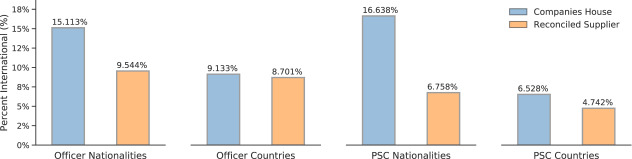

### Board interlock in government suppliers

Data linkage with Companies House allows us to identify company directors and secretaries with custom UIDs based on an anonymised hash of other auxiliary information. This UID subsequently allows us to see which anonymised Officers are sitting on a multitude of different company boards-a topic not uncommon in the corporate governance [22] or network science literature [23]. For the first time, however, we are able to analyze this overlap with specific regard to those companies supplying the government. Within the dataset produced by centgovspend at the time of writing, we estimate that there are 3,298 individual companies (nodes) enjoying a minimum of one board interlock, with a total of 5,714 interlocks (edges) between them. In terms of the largest absolute number of board interlocks on an officer basis (i.e. non-unique per company), by far the largest number of ‘edges’ are enjoyed by Ernst & Young Llp (4,266) and Deloitte Llp (878): an unsurprising finding given the convention for reciprocal interlocking of chief executive officers in large (accounting) corporations [24]. Table 1 outlines the ten most central companies (ordered by eigenvector centrality) in the Giant Component (the largest connected group of companies), specifically identifying Babcock International: a multinational corporation specializing in managing complex assets and infrastructure which is also recognized as one of the government’s 30 ‘Strategic Suppliers’ [25].

**Table 1: Centrality in the Giant Component:** **Table Notes:** Pay (£m) relates to total summed value of payments, while Pay (#) refers to total number of payments. Eigen represents the eigenvector centrality measure, Degree represents degree centrality, Close represents closeness centrality and Betwn represents betweeness centrality. Table is ordered by Eigenvector centrality.

Number	Name	Pay (£m)	Pay (#)	Eigen	Degree	Close	Betwn

SC099884	Babcock Support Services Ltd	173.98	359	0.216	0.143	0.241	0.051
9729579	Fixed Wing Training Ltd	25.35	2	0.215	0.137	0.243	0.133
3493110	Babcock Land Ltd	397.44	632	0.214	0.132	0.24	0.039
9329025	Babcock Dsg Ltd	1169.38	585	0.214	0.132	0.24	0.039
3700728	Flagship Fire Fighting Training Ltd	3.05	2	0.214	0.132	0.24	0.039
8230538	Babcock Civil Infrastructure Ltd	0.54	12	0.212	0.126	0.203	0.002
3975999	Cavendish Nuclear Ltd	1.52	14	0.212	0.126	0.203	0.002
SC333105	Babcock Marine (Rosyth) Ltd	51.73	540	0.211	0.132	0.205	0.07
6717269	Babcock Integrated Technology Ltd	91.27	505	0.211	0.126	0.203	0.006
2562870	Frazer-Nash Consultancy Ltd	19.15	246	0.209	0.121	0.203	0.004

## Discussion

This paper first surmises the ‘Open Data’ landscape within the United Kingdom and outlines and implements functionality to generate large datasets of public procurement data for analysis. It provides three prototype examples: each of which could be expanded into an independent body of work. In the following subsections we turn to the future and consider what this work makes possible, and what is required from a data origination perspective moving forward.

### Impact: Suggestions for Further Work

Making this library publicly and freely available makes all civic technologists, transparency advocates and enthusiasts, academic analysts and private sector suppliers able to explore a multitude of directions and generate their own context regarding the previously disparate data. First, it facilitates the future creation of an interactive dashboard of the reconciled dataset. This is important for the specific reason that the entire purpose of making the data available in the first instance was to inspire a generation of ‘armchair auditors’ as envisaged by David Cameron, and this has failed to fully materialize until now. Second, the library also enables the potential for ‘hackathons’ focused on civic technology and transparency. Third, it provides a database for Non-Governmental Organizations (NGO) and think tanks such as Transparency International and the Institute for Government who are already working in this space [3, 19]. It is of interest to such organizations due to its ability to isolate data on extremely large redacted payments and enforce commercial discipline by trawling for blacklisted suppliers and disqualified company directors. At present, we estimate that there are £120m of redacted payments and £178bn of nondescript supplier entities aggregated as ‘various’ remaining in the data. The supporting functions enable a significant number of new academic questions relating to company structure and ownership, such as board overlap in the set of reconciled companies and in Companies House more generally. It also enables the more specific calibration of models in the field of public economics and the estimation of marginal returns to investment in specific industries, or to further analyze national disparities of spending on public services [26]. There are also a multitude of commercial applications where the software can be utilized within the bidding process of Small and Medium Enterprise (SME) companies.

### Recommendations: The Data Origination Process

The limitations of the data are alluded to above and are discussed elsewhere [3, 9]. However, we surmise and order in importance what we view as the most pressing issues, and provide corresponding recommendations below:

1. **Issue:** There are no UIDs for suppliers, buyers, individual transactions or contracts.
  **Recommendation:** Utilize registers like Companies House and the Charity Commission.
2. **Issue:** The data is frequently provided in an inconsistent format.
  **Recommendation:** Incentivise the use of Resource Description Framework (RDF) and 5* data.
3. **Issue:** Data is not timely, and in some cases is missing for several years.
  **Recommendation:** Legislate sanctions for departments which repeatedly fail to comply.
4. **Issue:** Fields and their definitions vary from file to file.
  **Recommendation:** Renew guidance and training on production of “Spend over £25,000” data.
5. **Issue:** Data provision is decentralized across multiple domains.
  **Recommendation:** Charter a unit such as the Government Digital Service to curate an API.

There will likely be additional issues (such as an inability at present to ever truly identify sub-contractors), many of which are solved in part by centgovspend. However, the most pressing issue is the lack of UIDs for suppliers which would remove the need for approximate string matching based techniques to facilitate reconciliation to registers such as Companies House and the Charity Commission. This technological improvement will singularly unlock vast amounts of potential, and represents a commitment outlined in both Section 4.6 of the Anti-Corruption Strategy 2017–2022 [27], and on the front page of the draft of the National Action Plan for Open Government 2018–20 [28]. However, it remains to be seen how this will be enacted given the sporadic compliance to existing guidance [29], or how closely the data will represent “5^*^ Linked Open Data” (LOD) or utilize the Open Contracting Data Standard. As Theresa May noted in her re-affirmed commitment to Open Data in a letter to her Cabinet colleagues in 2017: “It is not enough to have open data; quality, reliability and accessibility are also required” [30].

## Conclusions

Current problems surrounding data origination do not change the value of the data when it is made available in an accessible format. The regularly maintained library described herein aims to act as an intermediate aggregation tool which also potentially inspires related work in an international context as well as making possible a multitude of commercial and inter-disciplinary academic contributions across a range of sub-fields. Our illustrative examples outline three academic prototypes of what is made possible, each of which being extensible into fuller bodies of work which are beyond the scope of this introductory paper. We envisage a burgeoning of public administration data science in future years which utilizes not only procurement data, but the wealth of information made available through the on-going ‘Open Data’ revolution.

## Supplementary Material

The paper is accompanied by a Github repository which also contains a Jupyter Notebook which details the analysis referred to herein. The library can be found at github.com/crahal.
